# Plasmonic CROWs for Tunable Dispersion and High Quality Cavity Modes

**DOI:** 10.1038/srep17724

**Published:** 2015-12-03

**Authors:** John J. Wood, Lucas Lafone, Joachim M. Hamm, Ortwin Hess, Rupert F. Oulton

**Affiliations:** 1 The Blackett Laboratory, Department of Physics, Imperial College London, London SW7 2AZ

## Abstract

Coupled resonator optical waveguides (CROWs) have the potential to revolutionise integrated optics, to slow-light and enhance linear and non-linear optical phenomena. Here we exploit the broad resonances and subwavelength nature of localized surface plasmons in a compact CROW design where plasmonic nanoparticles are side coupled to a dielectric waveguide. The plasmonic CROW features a low loss central mode with a highly tunable dispersion, that avoids coupling to the plasmonic nanoparticles close to the band-edge. We show that this low loss character is preserved in finite plasmonic CROWs giving rise to Fabry-Perot type resonances that have high quality factors of many thousands, limited only by the CROW length. Furthermore we demonstrate that the proposed CROW design is surprisingly robust to disorder. By varying the geometric parameters one can not only reduce the losses into dissipative or radiative channels but also control the outcoupling of energy to the waveguide. The ability to minimise loss in plasmonic CROWs while maintaining dispersion provides an effective cavity design for chip-integrated laser devices and applications in linear and non-linear nano-photonics.

Since their inception coupled resonator optical waveguides (CROWs) have emerged as an exciting technology for the integrated optics community^1^. On-chip guiding of light allied to the control of dispersion offered by CROW designs have opened the door to a raft of interesting phenomena including slow light and enhanced light-matter interaction^2^,^3^,^4^,^5^,^6^,^7^ and over the last decade CROWs have been explored extensively with a view to applications in optical switching^8^, optical filters^9^, delay lines^10^, sensing^11^ and wavelength conversion^12^. Since the exploitation of CROWs has so far been mainly confined to applications within photonics they typically employ low-loss dielectric resonators with narrow line-widths to minimise the prospective losses; yet these devices are very sensitive to disorder through fabrication imperfections due to the high quality resonances required. A CROW composed of plasmonic resonators (or plasmonic CROW) is yet to be investigated.

In contrast to dielectric resonators plasmonic nanoparticles have broad resonances, enable sub-wavelength confinement of light and have high associated losses. For systems of directly coupled resonators these losses can be detrimental to the waveguiding properties as the energy of the incident wave is absorbed^13^,^14^. However, in side coupled CROW schemes^15^,^16^,^17^,^18^ only a fraction of the energy flows in/out of the resonator and no losses occur as the light travels between resonator sites through the dielectric waveguide. In other words, within a side-coupled CROW the modes of the resonators are hybridised with the underlying waveguide structure so the plasmonic losses can be minimal. Interestingly, aside from mitigation of plasmonic losses, hybrid plasmonic-photonic systems have been shown to exhibit phenomena such as extraordinary optical transmission^19^ and waveguide plasmon polaritons^20^.

Furthermore, within the side coupled CROW the broad resonances of the plasmonic nanoparticles are beneficial as they interact strongly with light over a large frequency range providing a large degree of tunability as the nanoparticles are gradually brought into resonance with the sharp band-structure. As we show in this article, this mechanism allows extensive dispersion control as well as the ability to tune the hybrid nature of the modes and thereby affect the various loss channels from the plasmonic CROW. A further advantage of the use of plasmonic resonators is that the physical size of potential plasmonic CROW devices can be significantly compacted as the resonator size is no longer a limiting factor.

In this letter we investigate a plasmonic CROW that evanescently couples cut-wire resonators to photonic TE waveguide modes of a dielectric slab (Fig. 1). By design the structure enables; control of the coupling strength^21^, easy tuning of the plasmonic resonance and simple integration with on-chip devices through the silicon-on-insulator (SOI) platform. This intrinsic link between the geometric parameters and the CROW properties allows for dispersion control of the low loss CROW band which avoids coupling to the plasmonic nanoparticles. Moreover, we find that finite length plasmonic CROWs sustain the avoided coupling to the nanoparticles giving rise to high quality (Q ∼ 4000) cavity modes with strong mode discrimination, which are particularly interesting given the otherwise inherently lossy nature of plasmonic systems. Importantly, the transfer of energy out of these modes can be manipulated by altering the geometry to allow increased outcoupling to the waveguide channel. Finally, the effect of structural disorder on the CROW cavity mode is considered and we find that the characteristics of the hybrid resonances are robust against fabrication imperfections, particularly in the case of small resonator-array detuning.

## Results

### Theory of Mode Hybridisation

**Figure 1:**
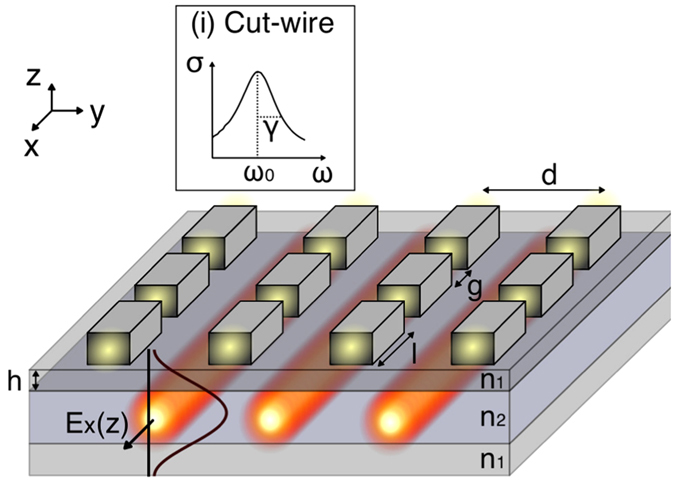
Schematic of the general geometry analysed in this paper. A dielectric stack waveguide of 3 layers, within the top layer there is a periodic array of metallic cut-wires. The parameters that define the structure are the refractive indices of the dielectric stack (alternating 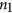, 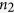, 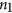), the array pitch, d, and the cut-wire segment length and gap length, l and g. The resulting CROW mode electric field profile (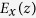) is represented showing the high concentration of fields within the central layer as well as the excited cut-wire resonators. The inset (i) illustrates the broad spectral response of a plasmonic cut-wire.

The photonic field is guided within the semiconductor slab as waveguide modes of a tri-layered slab with alternating refractive indices (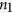, 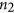, 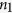). For the plasmonic nanoparticles we have used cut-wires due to their strong interaction with the TE waveguide modes and the ease with which their resonances can be altered. The cut-wires, located within the upper layer a distance *h* from the central layer (see Fig. 1), are composed of silver with 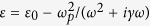. The Drude parameters, 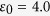, 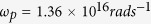, 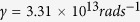 were chosen to fit the numerical data reported by Johnson and Christy^22^. Throughout this work we assume the refractive indices to be 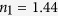 (e.g. Silica) and 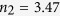 (e.g. Silicon) and a central layer width of 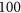 nm; whilst the two cladding layers are assumed to be semi-infinite in width. We note that the waveguide mode frequency at the edge of the first Brillouin zone (at 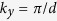) is governed by the array pitch, 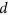, and can be expressed as 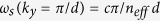, where 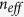 is the effective refractive index experienced by the waveguide mode.

The plasmonic modes of the cut-wires are dependent on both the material and geometry. The resonant frequency of the mode along the cut-wire axis, 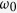, can be altered by changing either the lengths of the cut-wires segments, *l*, or the gaps, *g*; Fig. 1: inset i shows an example of the broad cut-wire resonance. Throughout the rest of this paper the cross-section is kept constant at 30 × 50 nm and 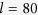
nm unless otherwise stated.

The dispersion of the hybrid mode is altered drastically when the wires are spaced such that the phase change of the waveguide mode between resonators is an integer multiple of 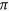. These effects are achieved since the three individual modes (two photonic and one plasmonic) are degenerate at the edge of the first Brillouin zone (FBZ); in other words the structure is tuned such that 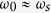. This resonant coupling between the array of cut-wires and the propagating photonic modes has been described as the waveguide plasmon polariton and has been shown to produce many interesting results including ultra-narrow and highly dispersive transparency bands^23^, slow light dispersion^24^, enhanced transverse magneto optical Kerr effect^25^ and enhanced generation of light^26^.

Before we proceed to the rigorous numerical calculations, we use a simple tight-binding model^23^,^24^ to calculate the expected behaviour of our system. By treating each cut-wire as a damped harmonic oscillator (*p*) driven by a time harmonic electric field (*E*) we can use the harmonic oscillator equation of motion as^27^


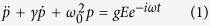


where g is the coupling between the field and the oscillator. There are three separate channels by which the excited oscillator can decay, emitting either out of the array or back into the waveguide and absorption losses within the oscillator. By splitting the losses 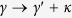 to include decay of the mode back into the waveguide (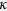) we can write the scattered field into the waveguide as 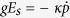. This allows the scattered field to be written as


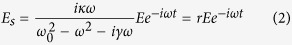


where *r* is the reflection coefficient. As the waveguide scattering has no preferential direction the transmission coefficient is given by 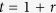 and the total transmission and reflection are calculated as 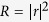 and 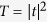.

**Figure 2:**
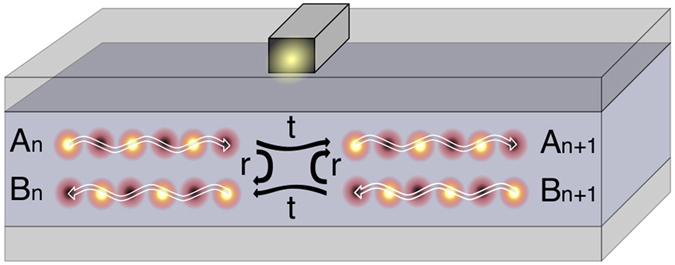
Schematic of the transfer matrix formalism detailing the scattering channels coupling the incoming and outgoing waves on either side of the cut-wire resonator for a single unit cell. The waveguide modes enter the unit cell, with amplitudes 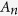 and 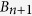, and propagate towards the cut-wire. At the cut-wire a fraction, 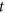, is transmitted and a fraction, 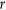, is reflected and they then propagate out of the cell, with amplitudes 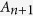 and 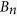.

Figure 2 shows how the waveguide modes incident on a cut-wire are transmitted and reflected within a single period or cell of the structure. Waveguide modes (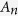 and 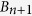) enter the cell from the left and right, then propagate between neighbouring cut-wires acquiring a phase, 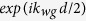, where 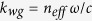. When the modes are incident upon the cut-wire they are reflected, transmitted or they can be absorbed through the oscillator losses (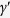). Propagating the amplitudes along the unit cell induces a phase, 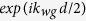, before the modes exit (as 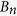 and 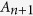). This can be written


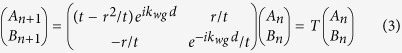


We note here that this is of the same form as reported in literature for similar side-coupled resonator systems^15^,^28^,^29^. To calculate the band structure we apply the Bloch conditions: 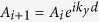 and 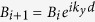. Upon inspection it is apparent that the eigenvalues of 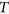 must be equal to the Bloch vectors 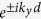. By writing 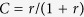, and solving for the eigenvalues we acquire the dispersion relation for such a structure as:


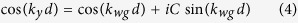


We have fitted this model to the numerical calculations that follow by varying the harmonic oscillator parameters (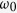, 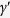 and 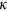).

### Dispersion Bands and Tunability

**Figure 3:**
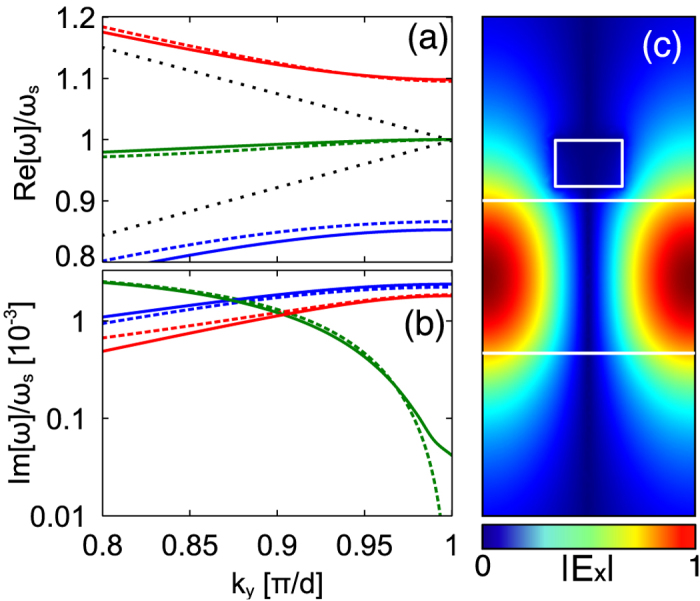
Dispersion relation for an infinite array of cut-wires with 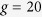
nm, corresponding to a single cut-wire resonance of 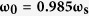. (**a**) The real part of the dispersion band and (**b**) the imaginary part of the dispersion band for numerical calculations (solid lines) and theory (dashed lines). Also shown are the bare waveguide modes (dotted black lines). (**c**) The electric field profile, 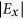, for a single unit cell in the y–z plane for the central dispersion band (green) at the band edge, 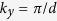. The colour scale ranges from blue (minimum) to red (maximum).

The modes resulting from hybridization between the cut-wire array and the slab waveguide are illustrated by the solid lines in Fig. 3a, where the pitch and cut-wire gaps were chosen to be 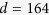
nm and 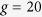
nm, respectively. (All simulations were performed using COMSOL.) As expected a large splitting is observed due to the coupling of the waveguide mode to the localized surface plasmon resonance. However, a flat central CROW band also emerges within the bandgap. Remarkably, this central mode is composed entirely of forward and backward propagating waveguide modes, with very little involvement from the cut-wires particles. This is evident from the electrical field distribution of the central mode (green) at the edge of the FBZ, Fig. 3c, which shows that the cut-wire sits in the node of the electric field, effectively unexcited. The lack of plasmonic characteristics in this central mode suggest low loss, which is confirmed by the small imaginary part of the dispersion relation (Fig. 3b). On the other hand, at 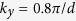 the loss of the central band is much higher; here the CROW mode is dominated by the cut-wire resonance. Since the low loss characteristics only occur for this central band near the FBZ edge, we focus the following discussion on this band.

The simple transfer matrix theory used to describe the system provides a good match to the band structure predicted by the rigorous calculations. The small discrepancies between the two descriptions originate from the influence of higher order waveguide modes in the simulation, which are not considered in this 3-band transfer matrix model. Indeed, the structure guides higher-order TE slab waveguide modes, which perturb the band structure through their interaction with the cut-wire resonances. Meanwhile, the transfer matrix theory breaks down in its prediction of the central mode’s loss at the edge of the FBZ. The discrepancy arises from the finite size of the cut-wire resonator, whereas the transfer matrix model assume point-like dipoles. Indeed, the loss of this central band must be non-zero due to a residual overlap of the cut-wires with the fields around the central mode’s nodes. We note that the loss is sensitive to the cross-section of the cut-wires and could thus be minimized further.

It is important to note that the central dispersion band changes character as 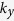 is varied; at 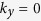 the band is predominantly the cut-wire plasmonic mode whereas at 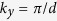 it is solely composed of waveguide modes. This dramatic change in character allows for the possibility that its shape can be manipulated. While the band’s position is fixed at the band edge with the frequency specified by the pitch of the array, 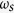, the cut-wire resonance determines the dispersion for 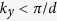. Thus by varying the frequency of the cut-wire mode 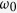 whilst keeping 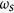 constant the shape of the central dispersion band can be tuned.

**Figure 4:**
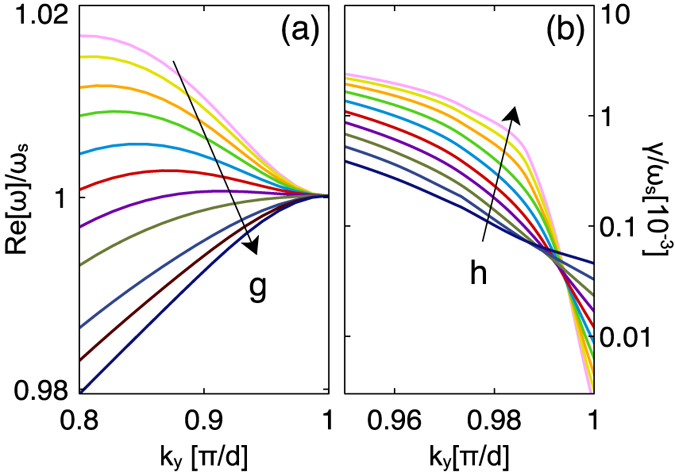
Tuning of the dispersion relation. (**a**) Real part of the central dispersion band tuned through the cut-wire resonance by changing the gap-size from 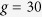
nm (pink line) to 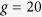
nm (dark blue line) in 1 nm increments. (**b**) Imaginary part of the central dispersion band tuned by changing the buffer height from 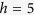
nm (dark blue line) to 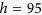
nm (pink line) in 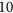 nm increments.

Figure 4a shows how the shape of the central dispersion band shifts as 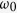 is changed over a small range. As expected, the shape of the real part of the dispersion band is varied and the sign of the gradient close to the band edge can be changed from positive to negative. It is therefore possible to engineer a band shape that is relatively flat over a range of k-values. As with traditional CROWs, this opens up the possibility of using such a structure for slow light applications. In contrast to the theoretical model, which predicts the possibility of a completely flat band (i.e. 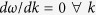), there are clearly limits in our ability to tune the *flatness* of the central band in the physical system arising from higher order waveguide modes which distort the band structure.

Despite the ability to engineer the real part of the dispersion relation, the imaginary part is not so easily changed. This is because the loss is mainly determined by the physical overlap of electric field near the nodes with the cut-wires, which cannot be modified by simply varying 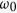. However, it is possible to engineer the imaginary part of the dispersion relation through other means. We can approach the theoretical mode loss limit by reducing the overlap between the cut-wire resonators and the waveguide modes. In Fig. 4b we reduce this overlap by increasing the height of the buffer layer, whilst maintaining the condition that 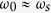. It can be seen that the loss of the mode is reduced, but only very close to the edge of the FBZ, where the hybrid mode is solely composed of waveguide modes. Counter-intuitively, away from the edge of the FBZ, the change in buffer height causes an increase in loss. This occurs because the larger spacer layer reduces the intrinsic coupling between waveguide mode and cut-wires. Thus the central mode maintains its cut-wire character further along the band than previously leading to the increase in mode loss. From a coupled mode theory perspective, the cross-over region for these two behaviours in Fig. 4b thus indicates the position where the plasmonic and photonic character of the central CROW mode is equal.

### CROW Cavity Modes

**Figure 5:**
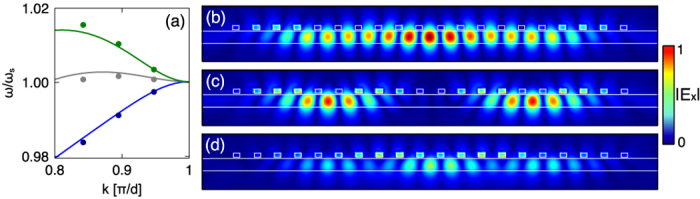
(**a**) Comparison of the dispersion relation (lines) and eigenmodes (dots) for arrays with 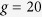
nm (blue) 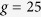
nm (grey) and 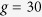
nm (green). The eigenmodes are found for finite arrays with 20 cut-wires. (**b–d**) 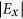 of the three highest order modes of an array 20 cut-wires with 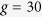
nm. From Equation 5: (**b**) mode 18, (**c**) mode 17 and (**d**) mode 16. The colour scale ranges from blue (minimum) to red (maximum).

We now turn our attention to finite arrays and their associated modes. The boundary between a region patterned with an array of resonators and the bare multilayer structure will inevitably induce reflections due to modal mismatch^30^. The flat nature of the central dispersion band and the associated high group index originates from the interference of forwards and backwards propagating modes in the patterned region, which cannot be matched to the scattered modes of the bare waveguide. Thus, a finite array of cut-wires on top of the multilayer slab supports Fabry-Perot type resonances. As with any Fabry-Perot resonator the corresponding resonances coincide with photon energies that cause an integer multiple of 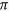 phase change across the structure. Taking the beginning and end of the structure to be the centre of the first and last cut-wire the expected k-values should then be given as


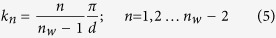


where 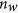 is the number of cut-wires in the array. By assigning the appropriate wavevector we can compare these modes of a finite structure with the dispersion bands of the infinite structure. A comparison is shown in Fig. 5a for modes 18, 17 and 16 of a 20 wire array for three different cut-wire resonances. There is a good agreement between the numerically determined eigenfrequencies of the finite modes and their mapping to the infinite band structure using Equation (5). The corresponding field distributions for modes 18, 17 and 16 of the 20 wire structure are shown in Fig. 5b–d. Importantly, the general field distribution of the infinite array from Fig. 3c is retained by these modes, since the field hotspots are located predominantly inside the waveguide, avoiding the cut-wire resonators. However, in contrast to the infinite case, the phase change across each unit cell is not sufficient to preserve this field profile throughout the array. As such, the mode gradually de-phases as it crosses the array leading to regions where the cut-wires are excited. At these points nodes appear in the electric field distributions and hence the modes appear to have standing wave type envelope functions.

The utility of plasmonic CROW cavities depends on how well the various cavity loss mechanisms can be controlled. These can be divided into radiation loss to free-space (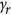), dissipative loss to the metallic resonators (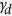), and waveguide coupling loss (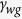). The parasitic cavity losses, 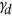 and 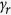 are enhanced in a finite array when cut-wire particles are excited. From Fig. 5b–d we have seen that excited cut-wires lead to nodes in the field distributions, thus the number of nodes in the field distribution indicates the parasitic loss rate of the modes. Hence, it is clear that the loss rates for each mode will be highly dependent on the mode number (as 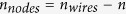). This is desirable trait of a laser cavity for example, since the least lossy mode will dominate the laser’s response^31^; in this case assuring the laser has a single longitudinal mode. In fact, for the array of 20 wires shown in Fig. 5b–d the Q-factors of the three modes are 544 (mode 18), 242 (17) and 195 (16), following approximately the ratio of the number of nodes; i.e. 1:2:3. Henceforth, we refer to the low loss mode as the fundamental mode of the laser cavity.

**Figure 6:**
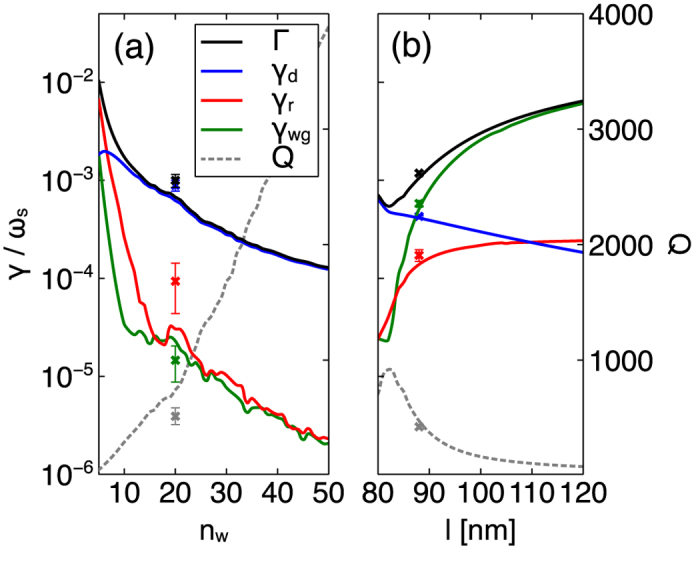
Change in the fundamental mode loss rates. The four rates are: 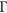, total losses, 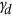, dissipative losses, 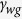, losses into TE_0_ waveguide mode and 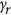, other radiative losses, and 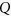 is the Q-factor. (**a**) Change in the loss rates with increasing number of wires in the array; the results shown are for an array of wires with 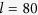
nm and 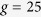
nm. (**b**) Change in the loss rates with an increasing cut-wire detuning; the results shown are for an array of 20 wires and 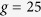
nm. The effect of disorder is shown by the crosses (mean) and error bars (standard deviation), averaged over 10 simulations for 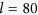
nm (a) and 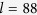
nm (**b**).

Before we focus on controlling the various sources of loss in the cavity, let us first focus our attention on how the number of wires affects the fundamental mode losses, shown in Fig. 6a, where the Q-factor for each array is also plotted. Remarkably, despite the fact that silver is a lossy material, the Q-factor of the fundamental mode continues to increase with the number of wires added to the array. This is slightly counter-intuitive as an increased amount of silver does not equal greater losses. Instead the displacement of the mode due to the increased number of cut-wires shifts the field hotspots further into the gaps, thus decreasing the spatial overlap between metal and field, working overall to decrease the modal loss and provide a higher Q-factor. In other words, the fundamental mode accumulates an increasing number of 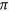 phase changes across the array (i.e. a higher wave-vector from eq. 5) and the mode progressively approaches the edge of the FBZ, where loss is minimized. Alternatively, one can see that as the mode gets larger, while the number of nodes at cut-wire sites is fixed, the proportion of light in metallic regions decreases, thus driving down loss; the mode profile becomes increasingly similar to that of Fig. 3c for the infinite array. At the same time the mode is acquiring an increasingly high group index introducing a further modal mismatch between the fundamental mode and the surrounding waveguide structure, which also decreases the waveguide loss. From Fig. 6 the Q-factor for an array of 50 cut-wires is ∼4000 but this is not an upper limit. Theoretically, modal losses would continue to decrease for larger arrays, tending towards the dispersion band loss at the edge of the FBZ (Q ∼ 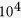). Here, loss could be further reduced through the resonator waveguide coupling (see Fig. 4) in order to attain even higher Q-factors.

The close correspondence in ratio of field node numbers with cavity mode order loss suggests that loss is dominated by the parasitic process; i.e. these cavity modes couple very little light to the waveguide. In fact, Fig. 6a shows clearly that dissipative loss dominates. Consequently, this particular CROW cavity design is plasmon-like. Although it is desirable to produce cavity modes with low losses, it is often useful to be able to tune the individual loss mechanisms since these direct the light as it leaves the cavity. For instance, if the cavity were to be used to construct a laser we would require the light to be emitted into the waveguide mode, for example. As we saw for the infinite array case, the dissipative loss and indeed the plasmon-photon character of the array is controlled by the cut-wire mode detuning. We have also analysed how the cut-wire mode resonance affects the various loss channels of the fundamental CROW cavity mode by increasing the length of the cut-wire segments, *l*, which in turn decreases the resonant frequency. By increasing *l* we can drastically increase the proportion of the mode energy that is transferred to the photonic waveguide mode (see Fig. 6b). This process not only enhances the waveguide coupling rate but decreases the dissipation rate by nano-particles. The reason for this increased coupling to the waveguide mode can be understood through the group index of the band structure. As the cut-wire mode frequency decreases below the array resonance the slope of the dispersion band becomes increasingly positive, thus increasing the group velocity of the fundamental mode. As a result, the mode mismatch between the cavity mode and that of the bare waveguide mode is reduced allowing for more efficient outcoupling of the mode out of the array to the point where radiation loss dominates, albeut with a reduced Q-factor. In this case, we can view this plasmonic CROW design as being photon-like and Q-factors in excess of 100 can be achieved with waveguide radiation loss accounting for at least 87% of cavity loss.

A major limitation in realising many of the initially suggested applications of CROWs is the high sensitivity to disorder^32^,^33^,^34^, in particular to that of the inter resonator spacing^35^. Many of these applications were deemed impossible or their efficiency was dramatically reduced as a result of unavoidable fabrication imperfections. In order to assess the viability of our system to proposed devices we have performed a number of simulations where the resonator spacing is subjected to random disorder in the range of ±1 nm and ±2.5 nm, respectively. The chosen values are within the stage position accuracy of commercially available EBL machines and should be achievable in practice. The results for the 2.5 nm fluctuations are presented as error bars in Fig. 6, where the crosses represent the mean value of the decay rates and the error bars are 1 s.d. long, averaged over 10 different random distributions.

It is well known that extrinsic losses due to disorder becomes more prominent in slow light systems as the group index increases. Therefore we have considered two separate resonator detunings (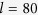 nm and 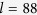
nm). The results for the 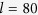
nm detuning, Fig. 6a (when resonator loss is dominant), show that the overall loss rates are increased and the Q-factor is reduced by 15–25% (±1 nm) and 30–40% (±2.5 nm). In contrast, for a slight detuning of 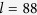 nm, Fig. 6b (when losses to the waveguide are comparable to resonator loss), the structure demonstrates far greater robustness; again the loss rates are increased but overall the Q-factor is only reduced by 1–3% (±1 nm) or 3–7% (±2.5 nm). For both detunings, the channel most affected by the disorder in the resonator position is the radiative decay rate. These results reveal a high level of robustness to disorder, particularly in the case of the slightly larger detuning.

The plasmonic CROW sustains Fabry-Perot type modes that reside mainly within the central layer of the photonic waveguide. Due to the steep loss dispersion of the structure there is strong mode discrimination and the fundamental mode is the least lossy. By increasing the number of cut-wires in the array it is possible to create Q-factors in excess of several thousand and although we have only shown results for sizes of up to 50 cut-wires there is no reason that these Q-factors could not be further increased with larger arrays. Finally, we have also seen how the modal loss to various channels can be engineered through the detuning of the cut-wire resonance allowing for the efficient outcoupling of energy from the fundamental mode to the waveguide modes of the stack. Such cavity modes could be highly desirable for producing compact on-chip single mode lasing devices and demostrate robustness against disorder in the array period.

## Conclusions

We have demonstrated that side coupled plasmonic CROWs display dispersion bands with low loss modes that avoid the plasmonic nanoparticles. Due to the broad nature of the plasmonic resonance there is extensive control of the dispersion characteristics over a range of frequencies. Moreover, the losses of the central CROW mode are highly dependent on the coupling parameter and can be reduced at the band-edge through tuning of the geometry. Within finite plasmonic CROWs these low-loss modes are preserved in the form of Fabry-Perot type resonances that similarly avoid coupling to the plasmonic nanoparticles. As the losses primarily arise from the overlap of the Fabry-Perot modes with the plasmonic nanoparticles the modal loss strongly depends on the mode order. This leads to strong mode discrimination which could be utilised in plasmonic CROW cavity laser devices. Importantly the geometry of the plasmonic CROW allows for tuning of the quality of the cavity and also the individual decay channels. By increasing the array size dissipation limited quality factors in excess of 4000 can be achieved, and by detuning the plasmonic and structure resonances the energy can be efficiently outcoupled into propagating waveguide modes. Finally, we have shown that the CROW cavity demonstrates a surprisingly high robustness to fabrication imperfections. This unprecedented control over the characteristics of the cavity modes along with the easy to fabricate on-chip design make the suggested structure an ideal candidate for applications in semiconductor optoelectronics.

## Additional Information

**How to cite this article**: Wood, J. J. *et al.* Plasmonic CROWs for Tunable Dispersion and High Quality Cavity Modes. *Sci. Rep.* **5**, 17724; doi: 10.1038/srep17724 (2015).

## References

[b1] Yariv A., Xu Y., Lee R. K. & Scherer A. Coupled-resonator optical waveguide: a proposal and analysis. Opt. Lett. 24, 711–713 (1999). 10.1364/ol.24.000711 18073830

[b2] Scheuer J., Paloczi G. T., Poon J. K. S. & Yariv A. Coupled resonator optical waveguides, towards the slowing and storage of light. Opt. Phot. News 16, 36–40 (2005).

[b3] Mookherjea S. & Yariv A. Kerr-stabilized super-resonant modes in coupled-resonator optical waveguides. Phys. Rev. E 66, 046610 (2002). 10.1103/PhysRevE.66.046610 12443352

[b4] Smith D., Chang H., Fuller K., Rosenberger A. & Boyd R. Coupled-resonator-induced transparency. Phys. Rev. A 69, 063804 (2004).

[b5] Yanik M. & Fan S. Stopping and storing light coherently. Phys. Rev. Lett. 71, 013803 (2005).

[b6] Yanik M. & Fan S. Stopping light all optically. Phys. Rev. Lett. 92, 083901 (2004). 14995773 10.1103/PhysRevLett.92.083901

[b7] Morichetti F., Canciamilla A., Ferrari C., Samarelli A., Sorel M. & Melloni A. Travelling-wave resonant four-wave mixing breaks the limits of cavity-enhanced all-optical wavelength conversion. Nat. Comms. 2, 296 (2011). 10.1038/ncomms1294 PMC3112537 21540838

[b8] Vlasov Y., Green W. M. J. & Xia F. High-throughput silicon nanophotonic wavelength-insensitive switch for on-chip optical networks. Nat. Phot. 2, 242 (2008).

[b9] Little B. E. *et al.* Very high-order microring resonator filters for WDM applications. IEEE Photon. Technol. Lett. 16, 2263–2265 (2004).

[b10] Xia F., Green W. M. J. & Vlasov Y. Ultracompact optical buffers on a silicon chip. Nat. Phot. 1, 65–67 (2006).

[b11] Kurt H. & Citrin D. S. Coupled-resonator optical waveguides for biochemical sensing of nanoliter volumes of analyte in the terahertz region. Appl. Phys. Lett. 87, 241119 (2005).

[b12] Morichetti F., Ferrari C., Canciamilla A. & Melloni A. The first decade of coupled resonator optical waveguides: bringing slow light to applications. Laser and Phot. Revs. 6, 74–96 (2012).

[b13] Maier S. A., Kik P. G. & Atwater H. A. Observation of coupled plasmon-polariton modes in Au nanoparticle chain waveguides of different lengths: estimation of waveguide loss. Appl. Phys. Lett. 81, 1714–1716 (2002).

[b14] Maier S. A. *et al.* Local detection of electromagnetic energy transport below the diffraction limit in metal nanoparticle plasmon waveguides. Nat. Mater. 2, 229–232 (2003). 12690394 10.1038/nmat852

[b15] Xu Y., Li L., Lee R. K. & Yariv A. Scattering-theory analysis of waveguide-resonator coupling. Phys. Rev. E 62, 7389–7404 (2000). 10.1103/physreve.62.7389 11102100

[b16] Weiss O. & Scheuer J. Side coupled adjacent resonators CROW-formation of mid-band zero group velocity. Opt. Express 17, 14817 (2009). 19687960 10.1364/oe.17.014817

[b17] Heebner J. E., Boyd R. W. & Park Q. H. SCISSOR solitons and other novel propagation effects in microresonator-modified waveguides. J. Opt. Soc. Am. 19, 722–731 (2001).

[b18] Heebner J. E., Boyd R. W. & Park Q. H. Slow light, induced dispersion, enhanced nonlinearity, and optical solitons in a resonator-array waveguide. Phys. Rev. E 65, 036619 (2002). 10.1103/PhysRevE.65.036619 11909297

[b19] Ebbesen T. W., Lezec H. J., Ghaemi H. F., Thio T. & Wolff P. A. Extraordinary optical transmission through sub-wavelength hole arrays. Nature 391, 667–669 (1998).

[b20] Christ A., Tikhodeev S. G., Gippius N. A., Kuhl J. & Giessen H. Waveguide-plasmon polaritons: strong coupling of photonic and electronic resonances in a metallic photonic crystal slab. Phys. Rev. Lett. 91, 183901 (2003). 14611284 10.1103/PhysRevLett.91.183901

[b21] Davies P. M. Z., Hamm J. M., Sonnefraud Y., Maier S. A. & Hess O. Plasmonic nanogap tilings: light-concentrating surfaces for low-loss photonic integration. ACS Nano 7, 7093–7100 (2013). 23826806 10.1021/nn402432m

[b22] Johnson P. B. & Christy R. W. Optical constants of noble metals. Phys. Rev. B 6, 4371–4379 (1972).

[b23] Zentgraf T., Zhang S., Oulton R. F. & Zhang X. Ultranarrow coupling-induced transparency bands in hybrid plasmonic systems. Phys. Rev. B 80, 195415 (2009).

[b24] Ishikawa A., Oulton R. F., Zentgraf T. & Zhang X. Slow-light dispersion by transparent waveguide plasmon polaritons. Phys. Rev. B 85, 155108 (2012).

[b25] Kreilkamp L. E. *et al.* Waveguide-plasmon polaritons enhance transverse magneto-optical kerr effect. Phys. Rev. X 3, 041019 (2013).

[b26] Rodriguez S. R. K., Murai S., Verschuuren M. A. & Gomez Rivas J. Light-emitting waveguide-plasmon polaritons. Phys. Rev. Lett. 109, 166803 (2012). 23215111 10.1103/PhysRevLett.109.166803

[b27] Bohren C. F. & Huffman D. R. Absorption and scattering of light by small particles, Ch. 9, p228–229, (Wiley 1983).

[b28] Fa S. Sharp asymmetric lin shapes in side-coupled waveguide-cavity systems. Appl. Phys. Lett. 80, 908–910 (2002).

[b29] Yanik M., Suh W., Wang Z. & Fan S.; Stopping light in a waveguide with an all-optical analog of electromagnetically induced transparency. Phys. Rev. Lett. 93, 233903 (2004). 15601162 10.1103/PhysRevLett.93.233903

[b30] Momeni B. & Adibi A. Adiabatic matching stage for coupling of light ito extended bloch modes of photonic crystals. Appl. Phys. Lett. 87, 171104 (2005).

[b31] Feng L., Wong Z. J., Ma R. M., Wang Y. & Zhang X. Single-mode laser by parity-time symmetry breaking. Science 346, 972–975 (2014). 25414307 10.1126/science.1258479

[b32] Ferrari C., Morichetti F. & Melloni A. Disorder in coupled-resonator optical waveguides. JOSA B 26, 858–866 (2009).

[b33] Mookherjea S. & Oh A. Effect of disorder on slow light velocity in optical slow-wave structures. Opt. Lett. 32, 289–291 (2007). 17215948 10.1364/ol.32.000289

[b34] Mookherjea S., Park J. S., Yang S. H. & Bandaru P. R. Localization in silicon nanophotonic slow-light waveguides. Nat. Phot. 2, 90–93 (2008).

[b35] Hughes S., Ramunno L., Young J. F. & Sipe J. E. Extrinsic optical scattering loss in photonic crystal waveguides: role of fabrication disorder and photon group velocity. Phys. Rev. Lett. 94, 033903 (2005). 15698268 10.1103/PhysRevLett.94.033903

